# Ulcère de Marjolin

**DOI:** 10.11604/pamj.2013.14.98.2497

**Published:** 2013-03-12

**Authors:** Wafae Raffas, Badreddine Hassam

**Affiliations:** 1 Service de Dermatologie, CHU Ibn Sina, Université Med V, Souissi, Rabat, Maroc

**Keywords:** Ulcère de Marjolin, brulure, ulcération chronique, Ulcère, greffe, Marjolin ulcer, burn, chronic ulceration, ulcer, transplant

## Image en médicine

L’ulcère de Marjolin désigne la transformation maligne d’une cicatrice de brûlure ou de toute autre plaie ou ulcération chronique. Rare, son incidence est estimée à 2% des cas. Le type histologique prédominant reste le carcinome épidermoïde (75%). Les membres en particulier les plis de flexion sont le siège préférentiel en raison des contraintes mécaniques exposant à la ré-ulcération. La période de latence moyenne est de 30ans. Ces cancers sont caractérisés par leur agressivité locale, des métastases plus fréquentes et un risque de récurrence et une mortalité plus importants que les carcinomes épidermoïdes classiques. L’amélioration du pronostic nécessite non seulement un diagnostic et un traitement précoce, mais surtout une attitude préventive qui consiste en des greffes cutanées précoces et des soins réguliers de toute cicatrice de brûlure. Nous rapportons le cas d’une patiente âgée de 68 ans, sans antécédents notables, qui consultait pour une tumeur ulcéro-bourgeonnante du creux poplité gauche apparue deux ans auparavant sur une ancienne cicatrice de brûlure datant de plus de 30 ans. La patiente avait consulté devant l’extension de la lésion et l’apparition d’un saignement au moindre contact. Par ailleurs on palpait des adénomégalies inguinales supracentimétriques bilatérales non inflammatoires. La biopsie cutanée confirmait le diagnostic suspecté cliniquement en objectivant un carcinome épidermoïde différencié infiltrant. L’échographie ganglionnaire montrait des adénopathies inguinales gauches d’allure métastatique. Les TDM du membre et thoraco-abdomino-pelvienne ne montraient pas d’envahissement ostéo-articulaire ni d’autres localisations secondaires. Une exérèse large avec curage ganglionnaire suivie d’une greffe était réalisée sans récidive après 6 mois de recul.

**Figure 1:**
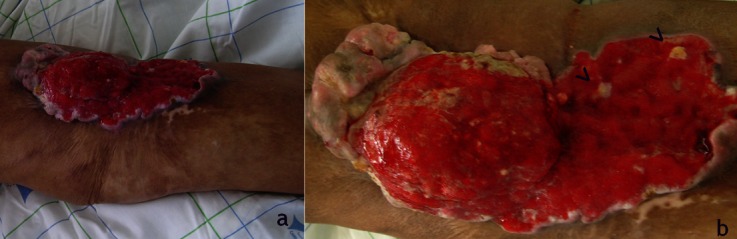
Ulcération indolore de 20cm de grand axe, à bordure surélevée indurée, à base infiltrée et à fond bourgeonnant couleur chair musculaire, avec issue de filaments de kératine ou du matériel nécrosé -“vermiottes”- à la pression (>). Noter la circulation veineuse collatérale périphérique.

